# Catheter-Guided Caudal Epidural Blood Patch for Refractory CSF Leak After Multiple Spine Surgeries: A Novel Case Report

**DOI:** 10.5812/aapm-164698

**Published:** 2025-11-09

**Authors:** Ebrahim Espahbodi, Nima Amiresmaili, Fahimeh Karimi

**Affiliations:** 1 Anesthesia, Critical Care and Pain Management Research Center, Tehran University of Medical Sciences, Tehran, Iran; 2 Preventative Gynecology Research Center, Shahid Beheshti University of Medical Sciences, Tehran, Iran

**Keywords:** Cerebrospinal Fluid Leak, Catheter-Guided Epidural Blood Patch, Caudal Approach, Fluoroscopy, MR Myelography, Postoperative Complication, Novel Intervention, Orthostatic Headach

## Abstract

**Introduction:**

Postoperative cerebrospinal fluid (CSF) leak is an uncommon but clinically important complication of lumbar spine surgery, typically presenting with orthostatic headache. When conservative management and dural repair are unsuccessful, image-guided interventions may be required.

**Case Presentation:**

We report a 48-year-old woman who developed a refractory CSF leak with disabling orthostatic headache following multilevel lumbar decompression. Despite initial conservative management and revision dural repair, symptoms persisted. Heavily T2-weighted magnetic resonance myelography (HT2W-MRM) localized the leak to the L4-L5 level. Conventional approaches such as re-exploration, lumbar drainage, or standard lumbar epidural blood patch (EBP) were relatively contraindicated because extensive postoperative fibrosis, adhesions, and altered epidural anatomy rendered direct lumbar access unsafe and technically impractical. The main intra-procedural challenges included navigating through scarred epidural planes and ensuring precise blood delivery to the leak site without risking additional dural trauma. Given the presence of postoperative fibrosis and technical inaccessibility via standard lumbar approaches, a fluoroscopy-guided caudal epidural blood patch (CEBP) was performed. A flexible epidural catheter was inserted through the sacral hiatus and advanced under real-time fluoroscopy to the identified leak site. Twenty milliliters of autologous blood were injected incrementally into the posterior epidural space. The patient experienced complete resolution of orthostatic headache and cessation of CSF leakage within 24 hours. At both two-week and two-month follow-up visits, she remained symptom-free, with no neurological deficits or recurrence. No procedural complications were observed.

**Conclusions:**

This case highlights the feasibility, safety, and clinical efficacy of catheter-guided CEBP performed under fluoroscopic guidance for treating complex, refractory CSF leaks in the early postoperative period. Importantly, it is one of the first reports to demonstrate this approach immediately following failed surgical dural repair, a scenario rarely documented in the literature. By integrating high-resolution MR myelography with precise catheter navigation, targeted therapy can be delivered effectively in anatomically altered spines where conventional techniques are contraindicated. Thus, this case uniquely illustrates how catheter-guided caudal access can serve as a novel, minimally invasive option in the early postoperative setting when direct lumbar access is no longer feasible.

## 1. Introduction

Cerebrospinal fluid (CSF) leak is an uncommon but clinically significant complication of spinal surgery or intervention. Despite its low incidence, it can cause spontaneous intracranial hypotension (SIH), which most often presents with orthostatic headache that worsens on standing and improves with recumbency (1). The underlying mechanism is CSF volume depletion, producing brain sag and meningeal traction that cause headache, often accompanied by visual, vestibular, or auditory symptoms such as nausea, dizziness, tinnitus, and neck stiffness (2). Cognitive slowing and impaired alertness have also been reported, further reducing functional capacity and overall quality of life in affected patients (3). The etiologies of CSF leak are diverse, ranging from iatrogenic injury (such as during lumbar puncture or spinal decompression) to spontaneous rupture of dural defects, which may be associated with connective tissue disorders, discogenic microspurs, or osteophyte complexes. Among postoperative causes, multiple spinal surgeries significantly increase the risk of CSF leakage due to cumulative dural fragility, epidural fibrosis, and anatomical distortion, all of which complicate both diagnosis and treatment (4).

Early recognition is essential, yet clinical presentation alone is often insufficient to localize the site of leakage. Historically, computed tomography (CT) myelography with intrathecal contrast has served as the gold standard for identifying the precise location of spinal CSF leaks, enabling targeted therapeutic approaches such as epidural blood patch (EBP) (5). However, CT myelography entails lumbar puncture and contrast administration, both of which may exacerbate existing leaks or be contraindicated in patients with reduced thecal sac volume or prior surgical hardware. In recent years, heavily T2-weighted magnetic resonance myelography (HT2W-MRM) has emerged as a powerful, non-invasive imaging modality. It provides high-resolution visualization of epidural fluid collections without the need for radiation or contrast, making it increasingly favored for both diagnosis and procedural planning in CSF leak management (6). However, access to such advanced imaging modalities may be limited in many clinical settings, which can affect diagnostic accuracy and subsequent procedural planning (1).

Management of CSF leaks is stepwise: Conservative measures such as bed rest, hydration, caffeine, and analgesia are first-line, particularly for low-output leaks, while persistent or high-output cases with significant intracranial hypotension require intervention (7). Among these, the EBP has become the cornerstone of invasive management. The mechanism of action is multifactorial: Injected autologous blood not only exerts a tamponade effect, increasing epidural pressure, but also promotes fibrin deposition and inflammatory sealing of the dural defect (8). Traditionally, EBP is performed via a lumbar interlaminar approach, which is anatomically direct and well-established. However, in the setting of previous laminectomies or spinal instrumentation, this approach may be limited by epidural adhesions, disrupted tissue planes, or technical inaccessibility (9). Conventional interlaminar EBP may be less effective in surgically altered spines, where adhesions or distorted planes restrict cephalad spread of blood (3). A catheter-guided approach enables controlled advancement and precise delivery at the leak level, and has been reported effective in refractory cases (7).

In such scenarios, a caudal epidural blood patch (CEBP) offers a highly effective alternative. Administered through the sacral hiatus, the caudal route permits widespread cranial distribution of autologous blood within the epidural space, enabling coverage of mid-to-upper lumbar and even thoracic levels while avoiding direct manipulation of prior surgical sites (10). Furthermore, this approach is associated with a lower risk of dural puncture or nerve root irritation, making it particularly suitable in anatomically distorted spines. The use of fluoroscopy adds significant value to the safety and precision of this technique. Under image guidance, real-time visualization allows accurate placement of the epidural catheter and verification of cranial spread, especially when combined with contrast-enhanced epidurography (11). The adjunctive use of a flexible epidural catheter further refines the procedure, permitting blood delivery precisely at or near the site of leakage, even when it is located at higher spinal levels. Numerous reports have documented the efficacy of catheter-directed caudal EBP in refractory cases, including those with ventral or foraminal leaks not amenable to blind lumbar injection (12). In parallel with procedural innovations, imaging modalities such as HT2W-MRM continue to evolve, playing a critical role not only in identifying the leak but also in planning the most appropriate route and level for blood patching. The HT2W-MRM has demonstrated diagnostic performance comparable to CT myelography and has been associated with symptomatic resolution rates exceeding 80% when used to guide targeted EBP placement (13).

Postoperative CSF leakage is uncommon but clinically consequential, with a reported incidence ranging from 0.3% to 17% depending on surgical complexity and revision status (3). Early recognition and timely management are essential to prevent complications such as meningitis or intracranial hypotension. Treatment typically follows a stepwise approach: Conservative measures including bed rest, hydration, caffeine, and analgesia are first-line, followed by surgical dural repair if leakage persists, and — when these fail — image-guided, minimally invasive techniques such as targeted EBP (9). In anatomically altered or postoperative spines, catheter-directed strategies enhance precision and efficacy by allowing targeted delivery of autologous blood directly at or near the leak site (7). As a result, it is increasingly integrated into clinical workflows as a first-line modality for both diagnosis and therapeutic planning.

In this context, we present the case of a 48-year-old woman with a refractory CSF leak following multilevel lumbar spine surgery, in whom traditional conservative measures and even direct surgical dural repair failed to resolve symptoms. Guided by HT2W-MRM imaging, a catheter-guided CEBP under fluoroscopic guidance was performed, targeting the identified leak at the L4-L5 level. The procedure resulted in immediate and sustained clinical improvement, highlighting the therapeutic potential of this approach in anatomically challenging cases. This case exemplifies the critical interplay between advanced imaging, catheter-based targeted delivery, and strategic route selection in optimizing outcomes in patients with complex postoperative CSF leaks.

## 2. Case Presentation

### 2.1. Patient Presentation

A 48-year-old woman presented with chronic low back pain and bilateral radicular symptoms. Neurological examination revealed sensory deficits and reduced reflexes, suggestive of lumbar nerve root compression. Magnetic resonance imaging (MRI) demonstrated multilevel disc herniations at L3-L4, L4-L5, and L5-S1, with significant spinal canal stenosis. With no prior history of spinal surgery or systemic illness, she was scheduled for lumbar laminectomy and discectomy in February 2022 to relieve neurogenic claudication and radiculopathy.

### 2.2. Surgical Procedure

Through a midline incision, decompression and discectomy were performed at L3-L5 using bilateral laminotomy and microsurgical techniques to preserve structures and reduce scarring. No intraoperative CSF leak or neural injury was observed. Hemostasis was achieved, the wound was closed in layers, and the patient was transferred to the post-anesthesia care unit (PACU) in stable condition.

### 2.3. Postoperative Complications

By postoperative day one, excessive drainage and orthostatic headache raised suspicion for a CSF leak. Conservative management (bed rest, hydration, caffeine, analgesics) failed over five days. Re-exploration revealed a small dural defect, which was repaired using monofilament sutures and fibrin sealant. A Valsalva maneuver confirmed no further leakage. The patient was again monitored postoperatively in the ward.

### 2.4. Further Intervention

**Figure 1.**
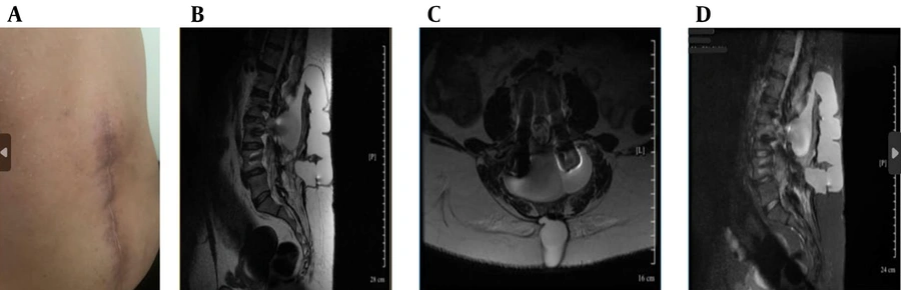
Clinical and magnetic resonance imaging (MRI) findings (A - D): A, postoperative lumbar scar with signs of cerebrospinal fluid (CSF) leak; B, sagittal T2-weighted MRI showing posterior epidural CSF collection; C, axial T2-weighted MRI showing hyperintense fluid posterior to the thecal sac; D, sagittal short tau inversion recovery (STIR) MRI showing cranially extending epidural CSF leak.

Despite repair, CSF leakage and orthostatic headaches persisted (Figure 1A). 

**Figure 2.**
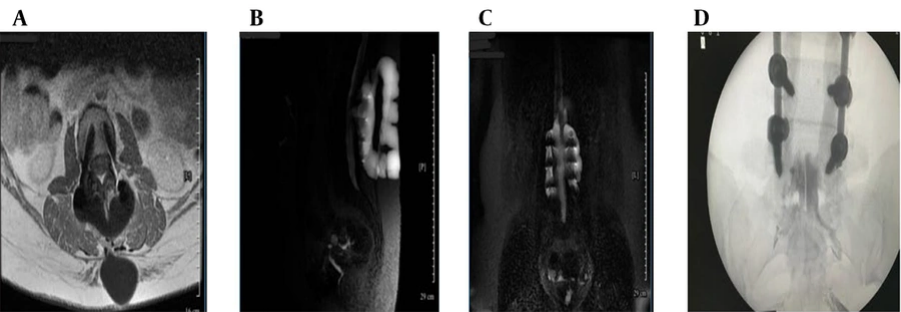
Magnetic resonance (MR) myelography and intervention (A - D): A, axial T1-weighted post-contrast magnetic resonance imaging (MRI) confirming enhancing epidural fluid; B, sagittal MR myelography showing contrast extravasation at L4-L5; C, coronal MR myelography confirming cerebrospinal fluid (CSF) tracking to sacral levels; D, fluoroscopic image showing catheter placement and cranial contrast spread.

Ongoing symptoms prompted further evaluation. The CT myelography was unavailable, so magnetic resonance (MR) myelography was used instead, revealing an active CSF leak at L4-L5 (Figure 1B - D and 2A), confirmed by contrast extravasation (Figure 2B and C). Given the failure of surgery and conservative therapy, an EBP was indicated. Direct lumbar access was avoided for three main reasons: (1) Postoperative fibrosis and adhesions at L4-L5, (2) risk of cephalad migration reducing efficacy, and (3) procedural difficulty associated with reoperated anatomy. A caudal approach was chosen for broader epidural spread, avoidance of manipulation of the surgical site, and minimizing procedural risks. Informed written consent was obtained from the patient for both the medical procedure and publication of this case report, in accordance with the Declaration of Helsinki. Ethical approval for this work was granted by the Research Ethics Committee of Tehran University of Medical Sciences (IR.TUMS.IKHC.REC.1404.152).

### 2.5. Epidural Blood Patch Procedure and Clinical Outcome

The patient was transferred to the operating suite for a catheter-guided CEBP under fluoroscopic guidance in sterile conditions and monitored anesthesia care. After standard positioning in the prone posture, with a pillow under the abdomen to optimize sacral access, the sacral hiatus was palpated and confirmed fluoroscopically. An 18-gauge Tuohy needle was introduced through the sacrococcygeal ligament and advanced under real-time fluoroscopic guidance into the caudal epidural space (Figure 2D). Following successful needle placement, a flexible epidural catheter was inserted through the Tuohy needle and advanced cranially within the posterior epidural space. Catheter navigation was performed under intermittent fluoroscopy with contrast injection to assess cranial spread and ensure accurate positioning. The catheter tip was advanced approximately 26 cm cranially from the sacral hiatus to reach the L4-L5 level, precisely corresponding to the dural defect identified on pre-procedural MR myelography. To facilitate controlled injection dynamics and improve maneuverability within the epidural space, approximately 20 cm of the catheter was trimmed prior to reinsertion. Epidurography confirmed accurate epidural positioning, demonstrating symmetric and progressive cranial dispersion of contrast without vascular opacification or inadvertent subarachnoid entry. Real-time anteroposterior–lateral fluoroscopic monitoring guided catheter navigation and ensured optimal placement despite postoperative anatomical distortion.

Before injection, the patient’s complete blood count and coagulation profile were reviewed to ensure normal clotting function, and autologous blood was used immediately after venipuncture to preserve optimal viscosity and coagulative potential. Subsequently, 20 mL of freshly drawn autologous venous blood was administered incrementally in 2 - 3 mL aliquots, with injection paused temporarily upon the onset of lumbar pressure sensation to prevent excessive epidural pressure. The procedure was performed by an experienced interventional pain specialist with extensive expertise in fluoroscopy-guided epidural interventions.

The catheter and needle were withdrawn without incident, and the puncture site was sealed with a sterile occlusive dressing. The patient was then placed in a supine position and monitored closely in the recovery unit for several hours. During this time, she remained hemodynamically stable and neurologically intact, with no back pain, radicular symptoms, or lower extremity weakness reported. Within 24 hours, the patient reported complete resolution of her orthostatic headache, which had previously been severe and functionally limiting. Inspection of the surgical wound confirmed complete cessation of CSF leakage, with a clean, dry surgical site. Neurological examination remained normal, and the patient noted a marked improvement in mental clarity, equilibrium, and overall well-being. No signs of post-dural puncture syndrome, urinary retention, or other complications were observed. Due to rapid and sustained clinical improvement, she was discharged on postoperative day two with recommendations for adequate hydration, gradual mobilization, and avoidance of activities that might elevate intracranial pressure.

At two-week and two-month follow-up visits, the patient remained asymptomatic, without recurrence of CSF leakage, orthostatic symptoms, or neurological complaints. Her functional recovery was complete.

This favorable clinical course highlights the effectiveness, safety, and durability of fluoroscopy-guided, catheter-guided CEBP in managing complex postoperative CSF leaks. Beyond technical execution, the success of the intervention relied heavily on accurate pre-procedural leak localization, precise catheter positioning, and real-time fluoroscopic guidance. Together, these elements allowed for targeted sealing of the dural breach in a surgically altered spine, reinforcing catheter-guided CEBP as a minimally invasive and highly effective therapeutic option when conventional approaches are not feasible.

## 3. Discussion

The patient demonstrated immediate and significant clinical improvement following the fluoroscopy-guided CEBP with catheter placement, characterized by complete cessation of CSF leakage and full resolution of orthostatic headache within 24 hours. The absence of procedural complications or adverse neurological events highlights the safety and feasibility of this technique in a postoperative spine, particularly when standard approaches are limited by scarring or altered anatomy (2). Her sustained clinical stability at two months post-procedure reinforces both the short- and intermediate-term effectiveness of catheter-guided CEBP in cases of refractory CSF leak (5).

Compared to conventional lumbar or transforaminal EBPs, the caudal route offers a distinct technical advantage in anatomically distorted or surgically altered spines (9). Catheter-guided CEBP has been reported to achieve precise delivery and higher efficacy in refractory cases (7). Moreover, caudal approaches have demonstrated safety and effectiveness in postoperative patients when conventional access is technically challenging (9). The sacral hiatus provides a natural entry point, and fluoroscopic guidance enables the advancement of a catheter cranially to deliver autologous blood directly to the vicinity of the identified dural breach, ensuring effective seal formation (1). This case particularly underscores the utility of combining fluoroscopy with flexible catheter navigation, which enhances procedural precision and minimizes the risk of suboptimal delivery or complications (10).

Comparative data in sacroiliac joint injections indicate that both ultrasound and fluoroscopy enhance clinical outcomes, with fluoroscopy favoring needle precision while ultrasound can shorten procedure time; this supports our fluoroscopy-guided choice in anatomically complex, postoperative settings (14). Direct lumbar approaches are frequently hampered by dense epidural fibrosis or prior instrumentation, increasing both technical complexity and procedural risk (6). These challenges highlight the clinical context in which alternative routes should be considered. In such scenarios, a catheterized caudal approach offers a significant therapeutic advantage by bypassing dense postoperative fibrosis, minimizing procedural bleeding risk, and enabling effective epidural blood delivery even when conventional interlaminar access is contraindicated (9). In contrast, the caudal method bypasses these challenges, allowing for widespread cranio-caudal dispersal of blood within the epidural space, maximizing contact with the affected segment (11).

Advanced imaging played a critical role in guiding management decisions. Due to unavailability of CT myelography, HT2W-MRM was utilized to localize the leak at L4-L5, clearly demonstrating ventral epidural contrast extravasation (12). Accurate leak localization is crucial for tailoring EBP delivery, especially in patients with multiple prior surgeries, and MR myelography has emerged as a powerful, radiation-free alternative (13). The synergistic application of imaging and targeted catheter-based therapy supports a personalized, anatomy-driven treatment paradigm (7). Fluoroscopy-guided caudal EBP is particularly indicated in patients with persistent CSF leaks or post-dural puncture headaches unresponsive to conservative measures, where precise epidural blood delivery significantly improves success rates, often exceeding 90%. However, it should be avoided in patients with active infection, uncorrected coagulopathy, or markedly elevated intracranial pressure, and performed cautiously when imaging guidance is unavailable (5).

In addition to clinical efficacy, the procedural efficiency — requiring minimal sedation, short procedural duration, and rapid discharge — further enhances the appeal of this technique in both inpatient and ambulatory settings. This is particularly relevant in healthcare systems emphasizing cost-effectiveness and patient-centered care. Although encouraging, the outcomes of a single case must be interpreted cautiously. Heterogeneity in dural tear location, CSF leak volume, and epidural compliance may influence success rates and warrant individualized planning (4). Larger prospective studies are needed to determine optimal blood volume, catheter depth, and imaging protocols to standardize care and improve reproducibility (3). It would also be valuable to examine patient-reported outcome measures, long-term recurrence rates, and comparative efficacy between caudal, lumbar, and transforaminal approaches in various clinical contexts (8).

This case contributes to the growing body of evidence supporting catheter-guided CEBP as a robust, adaptable, and clinically effective option for treating CSF leaks, particularly in patients with challenging spinal anatomy or prior surgical interventions. Beyond symptom relief, this approach offers strategic therapeutic advantages, including enhanced blood spread, reduced procedural trauma, and improved access to remote or ventral leak sites. As the body of literature expands, catheter-guided CEBP may redefine interventional standards, especially for post-surgical and recurrent CSF leaks. By integrating high-resolution diagnostic imaging, real-time fluoroscopic navigation, and catheter-based precision delivery, this approach embodies a minimally invasive yet maximally targeted solution to a complex neurospinal complication. It bridges the gap between radiologic localization and therapeutic execution, ensuring that intervention is not only anatomically precise but also functionally effective in restoring CSF dynamics and patient quality of life.

This case is noteworthy in that it represents one of the few reported instances where catheter-guided CEBP was successfully applied in the early postoperative period, following failed surgical dural repair. While the majority of published cases focus on spontaneous leaks or delayed interventions, this report highlights the feasibility and efficacy of an early, image-guided, minimally invasive approach in a surgically altered spine. The integration of MR myelography for precise leak localization and catheter-guided blood delivery via CEBP in the immediate post-laminectomy setting underscores a novel application of established techniques within a complex anatomical and clinical context. Despite the favorable short- and intermediate-term outcomes, the follow-up duration in this case was limited, and long-term efficacy or recurrence could not be evaluated.

### 3.1. Conclusions

This case highlights the feasibility, safety, and clinical effectiveness of catheter-guided CEBP in treating refractory CSF leak following multiple spinal surgeries. By integrating high-resolution MR myelography with fluoroscopy-assisted catheter navigation, the intervention achieved precise targeting of the dural defect and resulted in rapid and sustained symptom resolution without complications. Importantly, this case represents a novel clinical application of CEBP — performed in the early postoperative setting after failure of both conservative management and direct surgical dural repair. While most previous reports have focused on spontaneous or delayed leaks, this case demonstrates that catheter-guided caudal access can serve as a safe, effective, and minimally invasive alternative in surgically altered spines where conventional lumbar access is limited or contraindicated. The successful outcome reinforces the critical role of personalized interventional planning based on advanced imaging, anatomical considerations, and real-time procedural control. As such, this report contributes a meaningful addition to the evolving body of evidence supporting CEBP as a novel therapeutic strategy for complex CSF leak scenarios. Further prospective studies are needed to standardize technique, define patient selection criteria, and evaluate long-term outcomes. Until then, this case underscores the clinical relevance of catheter-guided CEBP as a novel, targeted solution in the management of challenging postoperative CSF leaks.

### 3.2. Limitations

Although this case provides valuable technical and clinical insights, it represents a single observation and therefore limits the external validity of the findings. Anatomical variability, dural defect location, epidural compliance, and the degree of postoperative fibrosis may all influence procedural success and reproducibility across different patients. The approach also relies on fluoroscopic guidance and operator proficiency, factors that may constrain its widespread adoption in resource-limited settings. While radiation exposure in this case was minimal, cumulative exposure should be considered in repeated procedures or multi-level applications. Another important limitation is the lack of microbiological confirmation beyond standard cultures; in postoperative CSF leaks, occult infection can mimic or exacerbate leakage, and polymerase chain reaction (PCR)-based assays have shown superior diagnostic sensitivity in such contexts (15). Finally, the short follow-up duration precludes assessment of long-term recurrence or durability of the seal.

### 3.3. Generalizability

Future multicenter prospective series and comparative trials are warranted to validate this minimally invasive approach, establish standardized procedural parameters, and define patient selection criteria for optimal outcomes.

## Data Availability

The dataset presented in this study is available on request from the corresponding author during submission or after publication. The data are not publicly available due to patient privacy and ethical restrictions.
